# Optimization and Application of Reflective LSPR Optical Fiber Biosensors Based on Silver Nanoparticles

**DOI:** 10.3390/s150612205

**Published:** 2015-05-26

**Authors:** Jiangping Chen, Se Shi, Rongxin Su, Wei Qi, Renliang Huang, Mengfan Wang, Libing Wang, Zhimin He

**Affiliations:** 1 State Key Laboratory of Chemical Engineering, Tianjin Key Laboratory of Membrane Science and Desalination Technology, School of Chemical Engineering and Technology, Tianjin University, Tianjin 300072, China; E-Mails: cjpjm@126.com (J.C.); shise@tju.edu.cn (S.S.); mwang@tju.edu.cn (M.W.); wanglb1@163.com (L.W.); enzyme@tju.edu.cn (Z.H.); 2 Collaborative Innovation Center of Chemical Science and Engineering (Tianjin), Tianjin 300072, China; 3 School of Environmental Science and Engineering, Tianjin University, Tianjin 300072, China; E-Mail: tjuhrl@tju.edu.cn

**Keywords:** localized surface plasmon resonance, optical fiber, silver nanoparticles, biosensors

## Abstract

In this study, we developed a reflective localized surface plasmon resonance (LSPR) optical fiber sensor, based on silver nanoparticles (Ag NPs). To enhance the sensitivity of the LSPR optical sensor, two key parameters were optimized, the length of the sensing area and the coating time of the Ag NPs. A sensing length of 1.5 cm and a 1-h coating time proved to be suitable conditions to produce highly sensitive sensors for biosensing. The optimized sensor has a high refractive index sensitivity of 387 nm/RIU, which is much higher than that of other reported individual silver nanoparticles in solutions. Moreover, the sensor was further modified with antigen to act as a biosensor. Distinctive wavelength shifts were found after each surface modification step. In addition, the reflective LSPR optical fiber sensor has high reproducibility and stability.

## 1. Introduction

Localized surface plasmon resonance (LSPR) is a nanoscale phenomenon based on a localized electromagnetic field around noble metal surfaces, which are sensitive to the surrounding refractive indices [1]. LSPR has many advantages, including label-free and real-time detection and tunable resonant wavelengths, which are controlled by careful adjustment of the size, shape and material of nanoparticles [2,3,4,5,6,7,8]. LSPR optical fiber sensors are based on the plasmon resonance of metal nanoparticles, coated on optical fiber surfaces, that are sensitive to changes in the surrounding medium. Accordingly, they may be used to detect label-free biomolecule interactions and to monitor the adsorption and desorption kinetics by continuous optical measurements [9]. Hence, LSPR optical fiber sensors have been widely used for biotin-streptavidin [10] and antigen-antibody interaction monitoring [11], metal ion detection, and other applications [12,13,14,15,16,17].

To date, many researchers have developed optical fiber LSPR sensors with various structures, e.g., the in-line transmission type [18,19,20] and terminated reflection type [21,22,23,24,25,26,27,28], a U-bend probe [29,30,31]. Compared to other structures, the terminated reflection type has shown greater merits. It possesses a much more efficient sensing area, and it does not require the use of flow-cells [32]. For this structure, a micro-fabricated silver mirror is usually coated at the end of the sensor probe to allow the incident light to travel twice through the sensing area without an increase in the sensing length. During the detection, the sensor probe is immersed into the testing solution without a need for flow-cells, which makes the optical setup much cheaper and simpler. Therefore, we selected the reflection type for further study, which includes fabrication and optimization of LSPR optical fiber sensor probes.

There are few studies concerning the preparation of LSPR optical fiber sensors based on silver nanoparticles (Ag NPs), although silver nanofilms have been proven to be much more sensitive to surrounding medium changes than other metal films [33]. Recently, Dutta *et al.* [34,35] produced a U-bend LSPR optical fiber sensor based on Ag NPs, but they did not further modify their LSPR sensor as a biosensor for detecting biomolecular interactions.

Therefore, the objectives of this study were to (1) fabricate a stable and sensitive reflective LSPR optical fiber sensor based on Ag NPs, (2) optimize the fabrication process, including two key parameters (the sensing length and the coating time), and (3) modify the surfaces of Ag NPs on the optical fiber, monitor the antigen-antibody binding process by continuous optical measurements, and observe the wavelength shifts during each modification step.

## 2. Experimental Section

### 2.1. Materials

Silver nitrate (AgNO_3_), 3-aminopropyl trimethoxysilane 97% (APTMS), 11-mercaptoundecanoic acid (MUA), 2-morpholinoethanesulfonic acid, *N*-(3-dimethylaminopropyl)-*N*′-ethylcarbodiimide hydrochloride (EDC), *N*-hydroxysuccinimide (NHS), and bovine serum albumin (BSA) were purchased from Sigma-Aldrich. All other chemicals were of analytical reagent grade. Human IgG (purified immunoglobulin, reagent grade), anti-human IgG (whole molecule, produced in rabbit) were purchased from the Wuhan Boster Biotechnology Company, China. Ultrapure water from a Milli-Q ultrapure water purification system (Millipore, Billerica, MA, USA) was used throughout the experiments. Multimode optical fibers of diameter 400 μm and NA = 0.22 were purchased from Ocean Optics.

### 2.2. Preparation of Silver Nanoparticles

The silver nanoparticles with a mean diameter of 30 nm chosen for this experiment were prepared as previously described [36]. Typically, an Ag NPs solution was synthesized by dissolving 0.026 g of sodium citrate in 50 mL of ethylene glycol. After the solution was stirred and heated to 90 °C, 0.012 g of AgNO_3_ was rapidly added, resulting in a color change from colorless to dark yellow after a few seconds. The mixture was continuously stirred and heated for approximately 10 min. The solution was diluted with 100 mL of deionized water and stored at 4 °C for future use.

### 2.3. Preparation of Ag NP-Based LSPR Sensor Probes

First, a polyimide-coating silica multimode optical fiber was cut into several 10-cm-long sections for use as sensor substrates. Both end surfaces of each fiber section were then carefully polished with polishing papers (using types of 12 μm, 3 μm, and 1 μm roughness, in sequential order, Ocean Optics). Each fiber section was left with a 2-cm unclad portion from the end to be used as a sensing area. The 2-cm portion was treated with 98% H_2_SO_4_ and 40% HF, respectively. The sensing area was then cleaned and hydrolyzed with Piranha solution (with a volume ratio of 7:3 of H_2_SO_4_:H_2_O_2_) for 30 min at 85 °C (it should be noted that this solution is extremely dangerous and needed to be handled with extreme care). Following that, the optical fiber was rinsed with deionized water, blow-dried with N_2_ and placed in an oven for 20 min at 110 °C.

For the Ag NP coating, the clean fiber was first immersed in a 10% solution of 3-aminopropyl trimethoxysilane (APTMS) in methanol for 1 h at 40 °C. The functionalized optical fiber was rinsed sequentially with ethanol and deionized water to remove unbound APTMS. After being blow-dried with N_2_, the optical fiber was placed in the oven for 20 min at 110 °C. Finally, the optical fiber was dipped into a Ag NPs solution for several hours. The incubated condition should be controlled at 4 °C to avoid random aggregation of Ag NPs on the fiber core surface. It has been shown that the aggregation of nanoparticles would significantly reduce the activity of nanoparticles, resulting in inferior performance of optical sensor probes [35].

To produce a reflective optical fiber sensor probe, a thin silver film was coated onto the end surface of the fiber probe using a chemical technique with Tollen’s reagent [37]. The preparation of Tollen’s reagents started with adding 2% ammonia solution drop-by-drop into 2 mL of 0.1 M AgNO_3_ solution. After the brown precipitate was totally dissolved, 1.4 mL of 0.8 M KOH was added to form a black precipitate in the solution. Subsequently, ammonia solution was added drop wise into the solution. Once the black precipitate was dissolved, Tollen’s reagent was acquired. Before silver mirror coating, the end surface of the fiber probe was dipped into a 0.2% tin (II) chloride dehydrate solution to sensitize the coating area. After rinsing thoroughly with deionized water and blow-drying with N_2_, the coating area was dipped into Tollen’s reagent. Subsequently, 0.4 mL of 0.25 M glucose was rapidly added; the fiber was then kept in the reagent for a period of 5 min to form the silver mirror. Afterwards, the fiber was removed from the reagent and rinsed with deionized water and blow-dried with N_2_. The sensor probe, now in its final form, was stored in deionized water at 4 °C for future use.

### 2.4. Functionalization of Sensor Probes

**Figure 1:**
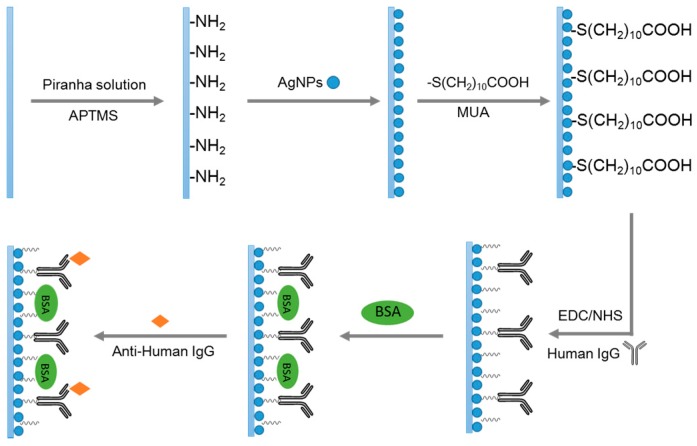
Schematic of bio-sensing approaches using the Ag NPs-based LSPR sensor probe developed in this work.

As shown in Figure 1, in order to create a Ag NP-based LSPR optical fiber biosensor for detecting the anti-human IgG, the as-prepared sensor probe was further functionalized (the human IgG was immobilized onto the sensor probe). The functionalization was carried out using a two-step procedure as follows. In the first step, we incorporated NHS-activated carboxyl groups onto the sensor probe. The sensor probe was immersed in a 10 mM MUA ethanol solution for 12 h to create a layer of carboxyl groups on the surface of the Ag NPs. The sensor probe was rinsed with ethanol and 0.01 M PBS (pH = 7.4) to remove unbound MUA. The sensor probe was then immersed into a mixed solution of 2 mM EDC and 5 mM NHS in 0.1 M MES buffer (pH = 6.0) with a volume ratio of 1:1 for 10 min to activate the carboxyl group of the MUA. After activation, the sensor probe was rinsed with PBS buffer. In the second step, we immobilized the human IgG onto the sensor probe surface and deactivated the residual NHS-activated carboxyl groups. The sensor probe was immersed in a 100 μg·mL^−1^ human IgG solution for 2 h to immobilize human IgG on the surface of the Ag NPs. After the immobilization, the sensor probe was immersed in 2 mg·mL^−1^ bovine serum albumin (BSA) solution for 1 h to suppress nonspecific binding. After that, the sensor probe was rinsed again with PBS to remove unbound BSA. Finally, the biosensor probe was created and stored in the PBS buffer at 4 °C for future use.

### 2.5. Monitoring of the Antigen-Antibody Interaction

After the functionalized procedure, the biosensor probe was ready to detect the specific anti-human IgG because the interaction between the anti-human IgG and the human IgG immobilized on the biosensor probe surface yields a change of a resonance wavelength in the reflection spectrum. To detect the anti-human IgG, the biosensor probe was immersed into the rabbit anti-human IgG solution to start the antibody-antigen reaction, and the reflection spectrum was monitored every five minutes.

### 2.6. Optical Setup and Data Processing

**Figure 2:**
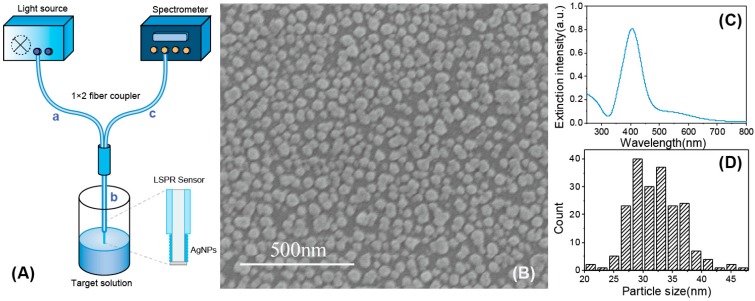
(**A**) Schematic of the experimental set-up used in this study; (**B**) An SEM image of AgNPs after being immobilized on optical fiber; (**C**) Absorption spectra of AgNPs solutions; (**D**) Histogram showing the corresponding particle size distribution of AgNPs.

The optical measurement set-up used in this experiment is shown in Figure 2A. In this optical system, a 1 × 2 fiber coupler was used to connect the sensor probe, the light source and the optical spectrometer. As illustrated in Figure 2A, the 1 × 2 fiber coupler has three light delivery paths. Part a and part c were connected to a tungsten halogen light source (HL-2000, Ocean Optics) and a spectrometer (HR2000+, Ocean Optics), respectively, and part b was connected to a homemade LSPR sensor probe by a splice bushing (21-02 SMA, Ocean Optics). Light emitted by the light source was coupled into the 1 × 2 fiber and travelled to the sensor probe. Due to the silver mirror at the end surface of the sensor probe, light was reflected back along the fiber and fed into the spectrometer. Thus, a computer could monitor, display, and record the optical signals measured by the spectrometer. The original data were analyzed using a homemade MATLAB code to identify the resonance wavelength from the obtained reflectance spectra over time.

## 3. Results and Discussion

### 3.1. Characterization of the Sensor Probe

The as-prepared Ag NPs were measured by UV-Vis absorption spectroscopy (TU-1810, Persee) over the wavelength range from 300 nm to 800 nm, as shown in Figure 2C. The peak located near 405 nm is typically observed for Ag NPs. As shown in Figure 2B, the Ag NPs immobilized on the surface of sensor probe were characterized by FE-SEM (S4800, Hitachi, Japan). According to the image, the particle diameter of Ag NPs was, on average, 30 nm (the counting histogram was shown in Figure 2D), and most of them were nearly spherical. In addition, most of the Ag NPs immobilized on the sensing surface were separated from each other. According to a previous report, the aggregation was a source of LSPR band broadening and wavelength red-shift [35]. Therefore, careful control of temperature of the self-assembly process to avoid the aggregation of nanoparticles was necessary in this experiment.

To determine the sensitivity of the as-prepared sensor probe to the surrounding refractive index, the sensor probe was immersed in a series of testing solutions of varying refractive indices, ranging from 1.33 to 1.40. The testing solutions were a series of different mass concentrations of sucrose solutions. The refractive indices of the sucrose solutions were measured by an Abbe refractometer. It is noticed that once the sensor probe was immersed in the testing solution, the spectrum changed immediately and stabilized quickly at the specific position corresponding to the refractive index of the testing solution.

**Figure 3:**
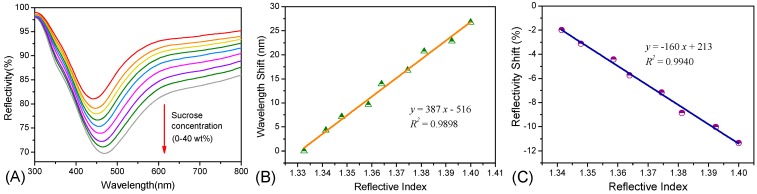
(**A**) The reflective spectra of the Ag NP-based sensors of sucrose solutions with different concentrations; (**B**) Changes in the LSPR wavelength shift to the refractive indices; (**C**) Changes in the LSPR reflectivity shift to the refractive indices.

The spectra acquired from the sensitivity test are illustrated in Figure 3A. With an increase in the refractive index, the relative reflectivity decreased accordingly. The LSPR wavelength showed a red-shift with an increase in the refractive index. This observation verified the sensitivity of the as-prepared sensor probe to refractive index changes to the surrounding medium.

The refractive index sensitivity was defined as follows [9,38]:

(1) $$\text{S}=\frac{\text{d}\lambda_{min}}{\text{dn}}$$

(2) $$\text{S}=\frac{\text{d}I_{min}}{\text{dn}}$$

where *S* represents sensitivity, *λ_min_* is the peak value in the reflectivity spectrum, *I_min_* is the optical intensity in the reflectivity spectrum and *n* is the refractive index value. The reflective peak wavelength and optical intensity observed from the sensor probe corresponding to each sensitivity test was recorded and calculated by a homemade MATLAB routine based on Equations (1) and (2). Figure 3B shows the shift in the LSPR wavelengths of the various sucrose solutions with different reflective indices. The sensitivity of the sensor probe to reflective indices was 387 nm/RIU, and the total peak wavelength shift was approximately 30 nm. Both the sensitivity and the total peak wavelength of the as-prepared Ag NP-based sensor probe were equivalent to those of another reported Ag NP-based fiber sensor probe with a different structure [34] and were much higher than those of other reported individual silver nanoparticles in solutions. Figure 3C shows the shift in the reflectivity of the sensor probe corresponding to the various sucrose solutions with different reflective indices. The sensitivity of the sensor probe was 160%/RIU in absolute value and the total optical intensity shift was approximately 12%.

According to a previous report [39], the resolution was also a key performance characteristic of an LSPR sensor and could be defined as the smallest change in the bulk refractive index that produced a detectable change in the sensor output. That is, the resolution of an LSPR sensor was related to the level of the standard deviation of noise of the sensor output. The formula was shown as follows:

(3) $${\text{r}}_{RI}=\frac{\sigma_{so}}{S_{RI}}$$

where *r*_RI_ represented resolution, *σ*_SO_ was the standard deviation of noise of the sensor output, *S*_RI_ was the refractive index value. As we used the HR2000+ spectrometer (Ocean Optics), the number of *σ*_SO_ of wavelength and reflectivity was 0.35 nm and 0.75%, respectively. Take the data from Figure 3 for example. 

As shown in Table 1, the *r*_RI_ based on wavelength shift was smaller than the *r*_RI_ based on reflectivity change, which was consistent with the compare result of refractive index sensitivity. With this method, we could compare the refractive index sensitivity of the two approaches: wavelength shift *versus* reflectivity change. Moreover, Cao *et al.* [12] had mentioned that wavelength-based technique was much more insensitivity to the interference of the surrounding environment. Therefore, we preferred to use the sensitivity based on wavelength peak shift rather than reflectivity in our following discussions. 

**Table 1:** Compare of the resolution of the as-prepared LSPR sensor based on wavelength shift and optical intensity change respectively.

	*σ*_SO_	*S*_RI_	*r*_RI_
Wavelength shift	0.35 nm	387 nm/RIU	9.04 × 10^−4^ RIU
Reflectivity change	0.75%	160%/RIU	4.68 × 10^−3^ RIU

### 3.2. Optimization of Sensor Performance

Prior to the biosensor applications of the sensor probe, two parameters, including the sensing length and the coating time, were optimized during the fabrication process.

**Figure 4:**
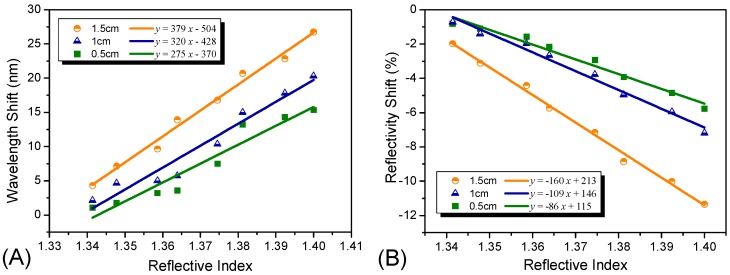
Refractive index sensitivities of the Ag NP-based sensors with different sensing lengths of 0.5 cm, 1 cm, 1.5 cm. (**A**) illustrates the wavelength shifts of the Ag NPs-based sensors with three different sensing lengths; (**B**) illustrates the reflectivity shift of the Ag NP-based sensors with three different sensing lengths.

To study the influence of the sensing length of the sensor probe, we prepared and tested a series of sensor probes with sensing lengths of 0.5 cm, 1 cm and 1.5 cm. The refractive index sensitivities are demonstrated in Figure 4, and the detailed reflective spectra can be seen in Figure S1 (Supplementary materials). As illustrated in Figure 4A, with an increase in the sensing length of the sensor probe, the refractive index sensitivity changed from 275 nm/RIU to 379 nm/RIU. The increase in the sensitivity may be due to the increase in the sensing area. A longer sensing length meant a larger sensing surface area; hence, more silver nanoparticles were immobilized on the sensing surface. Therefore, the interactions between the incident light and the nanoparticles would increase accordingly. In addition, according to Figure 4B, the optical intensity shift of the sensor probe increased with an increase in the sensing length, with a change from 5% to 11%, and the sensitivity increased from 86%/RIU to 160%/RIU. The optical intensity change is clearly evident and the sensitivity is high, indicating that both the peak wavelength and the optical intensity changed with the environmental refractive indices. What is more, the peak wavelength shift turned out to be much more sensitive.

**Figure 5:**
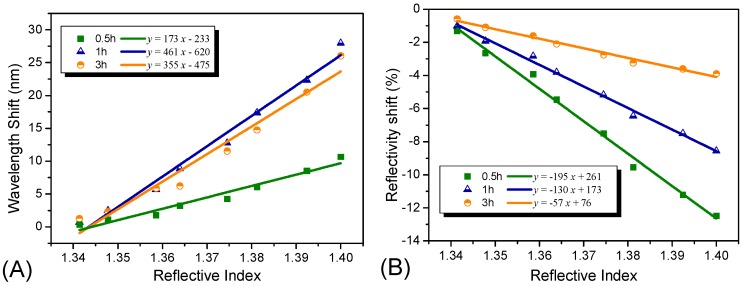
Refractive index sensitivities of the Ag NP-based sensors with different coating time of 0.5 h, 1 h, and 3 h. (**A**) illustrates the wavelength shifts comparison of the Ag NP-based sensors with three different coating times; (**B**) illustrates the optical intensity shifts comparison of the Ag NP-based sensors with three different coating time.

A similar experiment was carried out to investigate the influence of the Ag NP coating time on the sensitivity of the sensor probe. We produced a series of sensor probes with different coating times of 0.5 h, 1 h and 3 h. The results obtained are shown in Figure 5. As observed, with an increase in coating time, the sensitivity initially increased from 173 nm/RIU to 461 nm/RIU (Figure 5A). However, as the coating time further increased, reaching 3 h, the sensitivity decreased to 355 nm/RIU and a new plasmon resonance band was observed in the higher wavelength region from 600 nm to 800 nm in the reflectivity spectrum, as shown in Figure S2 (Supplementary materials). This observation appears to be different from a finding reported previously [40]. As predicted, there was an increase in the refractive index sensitivity relative to the decrease in the particle pair partner’s distances. As for this contradiction, we believed that the reason may be the aggregation of the absorbed nanoparticles. The appearance of a new plasmon resonance band further proves our assumption, which is inconsistent with that of other reported literature we mentioned above [35]. Hence, the total 1 h time was sufficient for the coating process using our approach. With longer coating times, a higher level of aggregation of the nanoparticles may occur, making it difficult to obtain a sensitive optical sensor probe. Moreover, we also studied the relationship between the optical intensity shift and the coating times; the results are illustrated in Figure 5B. With an increase in the coating time, the reflectivity decreased, and the sensitivity also decreased from 195%/RIU to 57%/RIU. The aggregation of the Ag NPs did not impart changes to the optical intensity.

### 3.3. Stability of the Sensor Probe

**Figure 6:**
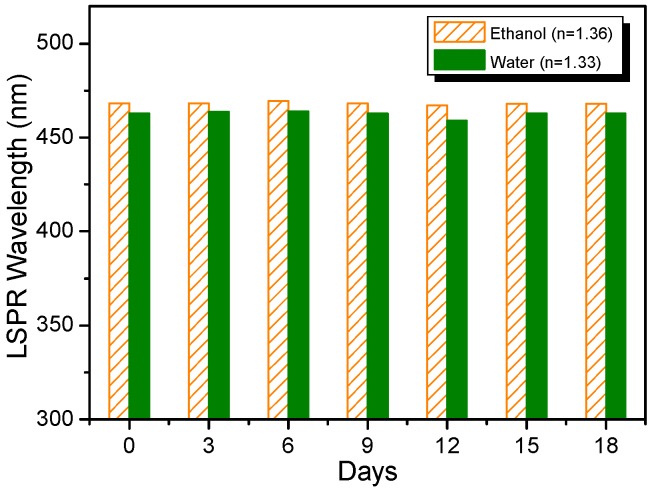
LSPR wavelength monitoring of the Ag NP-based sensor subjected in solutions with different refractive indices for a period of eighteen days.

To evaluate the stability of the sensor probes, a specific experiment was repeated every three days over a period of 18 days. For each test, the resonance wavelength of one freshly made Ag NP-based LSPR sensor probe was recorded by immersing the sensor probe into deionized water and then into ethanol. After each test, the sensor probe was stored at 4 °C in deionized water. As shown in Figure 6, only a small fluctuation in resonance wavelength for each testing solution was observed, indicating that storing the sensor probe in the deionized water at 4 °C was effective, as the Ag NP-based LSPR sensor probe persevered in this way had a good stability over the test period of at least 18 days.

### 3.4. Biosensor Applications (the Functionalization and the Bimolecular Interaction Monitoring of the Sensor Probe)

**Figure 7:**
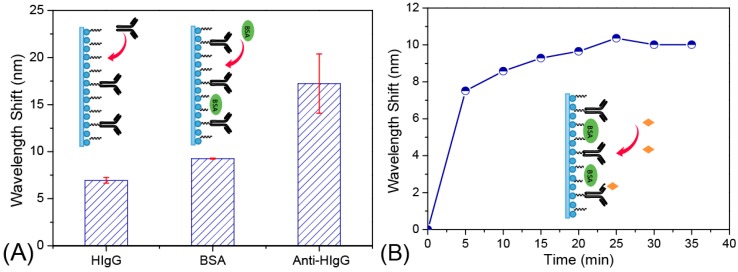
(**A**) Shifts of LSPR spectra of the Ag NP-based sensor after different stages of surface modification relative to the unmodified Ag NP sensor probe; (**B**) LSPR peak wavelength changes during the process of anti-human IgG immobilization on the Ag NP-based sensor surface over a period of 35 min.

After optimization of the sensor performances, we chose a Ag NP-based sensor probe with a sensing length of 1.5 cm and a coating time of 1 h for further biosensing applications, as illustrated in Figure 1. In this experiment, we used the interaction of human IgG and rabbit anti-human IgG as an analytical model for testing the application of LSPR sensors on the study of antigen-antibody interaction. Before the study of the antigen-antibody interaction, the Ag NP-based sensor probe was treated by a conventional EDC/NHS method to bind human IgG. The sensor probe was immersed in a PBS buffer (pH = 7.4) to obtain and record a stable spectrum, which was used as a baseline for further study of the peak wavelength shift of each modification stage. Afterwards, the sensor probe was immersed into a solution containing 200 μg·mL^−1^ human-IgG to begin the binding process. As shown in Figure 7A, the wavelength shift of each modification step was monitored (the error bars were the sample-to-sample deviation, N = 3). Each wavelength shift was based on the baseline obtained from the sensor probe immersed in the PBS buffer. After the sensor probe was functionalized with human-IgG, the wavelength shift was approximately 7 nm. To decrease the specific binding, the biosensor activated by EDC/NHS was then immersed in a BSA solution to cap the active sites. After the BSA blocking, a further wavelength shift of 3 nm was observed. The wavelength shift was not obvious, indicating that there were few bare NHS-activated carboxyl groups at the surface of sensor probe. Afterwards, the biosensor was immersed into 100 μg·mL^−1^ anti-human IgG to begin the antigen-antibody binding process. A further wavelength shift of approximately 8 nm was detected. The peak wavelength shift for each modification step was high enough to be observed indicating that the as-prepared optical fiber biosensor has potential application for monitoring antigen-antibody binding processes.

The functionalization process was monitored by recording the spectrum every 5 min. As shown in Figure 7B, a gradual red shift of LSPR peak wavelength was observed with an increase in the functionalized time. The gradual red shift was caused by the refractive index changing at the sensing surface caused by the immobilization of anti-human IgG on the Ag NP-based sensor surface. The shift of the spectrum eventually leveled out indicating that the antibody immobilization of the LSPR biosensor was finished.

## 4. Conclusions

In this work, we produced a Ag NP-based LSPR optical fiber biosensor and evaluated its refractive index sensitivity based on wavelength shift and reflectivity shift detection. We optimized the probe manufacturing process based on the sensing length of the sensor probe and the coating time of the Ag NPs. After the optimization process, the results showed that the refractive index sensitivity of the Ag NP-based LSPR sensor probe was 387 nm/RIU (wavelength shift) and 160%/RIU (reflectivity shift). Furthermore, we functionalized the sensor probe to demonstrate its use in bio-sensing applications. The fabricated biosensor probe could be used to monitor the interaction of an antigen and antibody. Compared to previously reported LSPR optical fiber sensors, we not only fabricated a stable and sensitive Ag NP-based reflective optical fiber sensor probe by a fast and simple method and optimized the fabrication process, we also used it to monitor the antigen-antibody binding process. With these systematic works, we demonstrated that the Ag NP-based reflective optical fiber sensor was able to be used as a biological sensor.

## Supplementary Files

Supplementary File 1
