# Supplementary material for: Optimization and Application of Reflective LSPR Optical Fiber Biosensors Based on Silver Nanoparticles
Source: Sensors (Basel). 2015 May 26;15(6):12205–17. doi: 10.3390/s150612205 (PMC4507690; doi:10.3390/s150612205)
Supplement: Supplementary File 1 [file sensors-15-12205-s001.pdf]

OPEN ACCESS
sensors
ISSN 1424-8220
www.mdpi.com/journal/sensors

Supplementary Information

Optimization and Application of Reflective LSPR Optical Fiber Biosensors Based on Silver Nanoparticles. *Sensors* 2015, *15*, 12205-12217

Jiangping Chen [1], Se Shi [1], Rongxin Su [1,2,*], Wei Qi [1,2,*], Renliang Huang [3], Mengfan Wang [1], Libing Wang [1] and Zhimin He [1]

[1] State Key Laboratory of Chemical Engineering, Tianjin Key Laboratory of Membrane Science and Desalination Technology, School of Chemical Engineering and Technology, Tianjin University, Tianjin 300072, China; E-Mails: cjpjm@126.com (J.C.); shise@tju.edu.cn (S.S.); mwang@tju.edu.cn (M.W.); wanglb1@163.com (L.W.); enzyme@tju.edu.cn (Z.H.)
[2] Collaborative Innovation Center of Chemical Science and Engineering (Tianjin), Tianjin 300072, China
[3] School of Environmental Science and Engineering, Tianjin University, Tianjin 300072, China; E-Mail: tjuhrl@tju.edu.cn

* Authors to whom correspondence should be addressed; E-Mails: surx@tju.edu.cn (R.S.); qiwei@tju.edu.cn (W.Q.); Tel./Fax: +86-022-2740-7599 (R.S.).

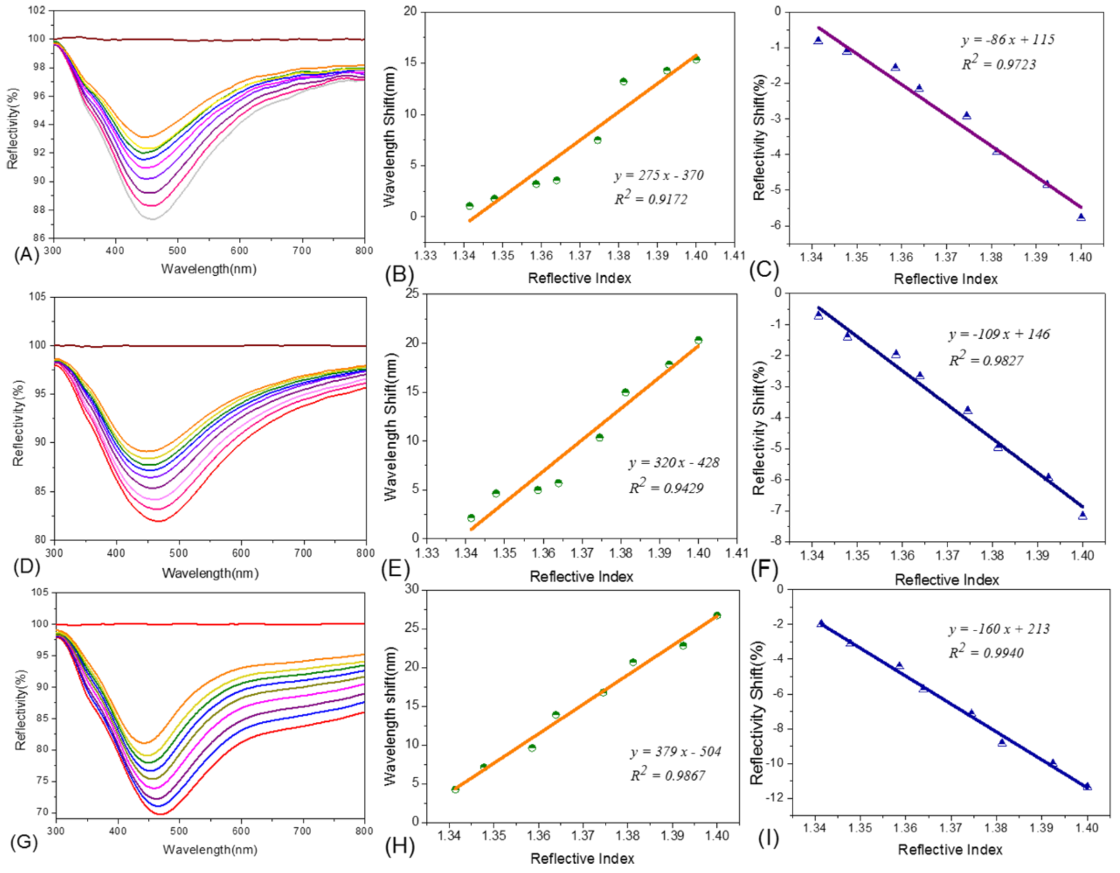

Figure S1. Refractive index sensitivities of the AgNPs-based sensors with different sensing length of 0.5 cm, 1 cm, 1.5 cm. (**A**,**D**,**G**) illustrate the reflective spectrum of the AgNP-based sensors with different refractive index solutions; (**B**,**E**,**H**) illustrates the wavelength shift comparison of the AgNP-based sensors with three different sensing lengths; (**C**,**F**,**I**) illustrates the optical intensity shift comparison of the AgNP-based sensors with three different sensing lengths.

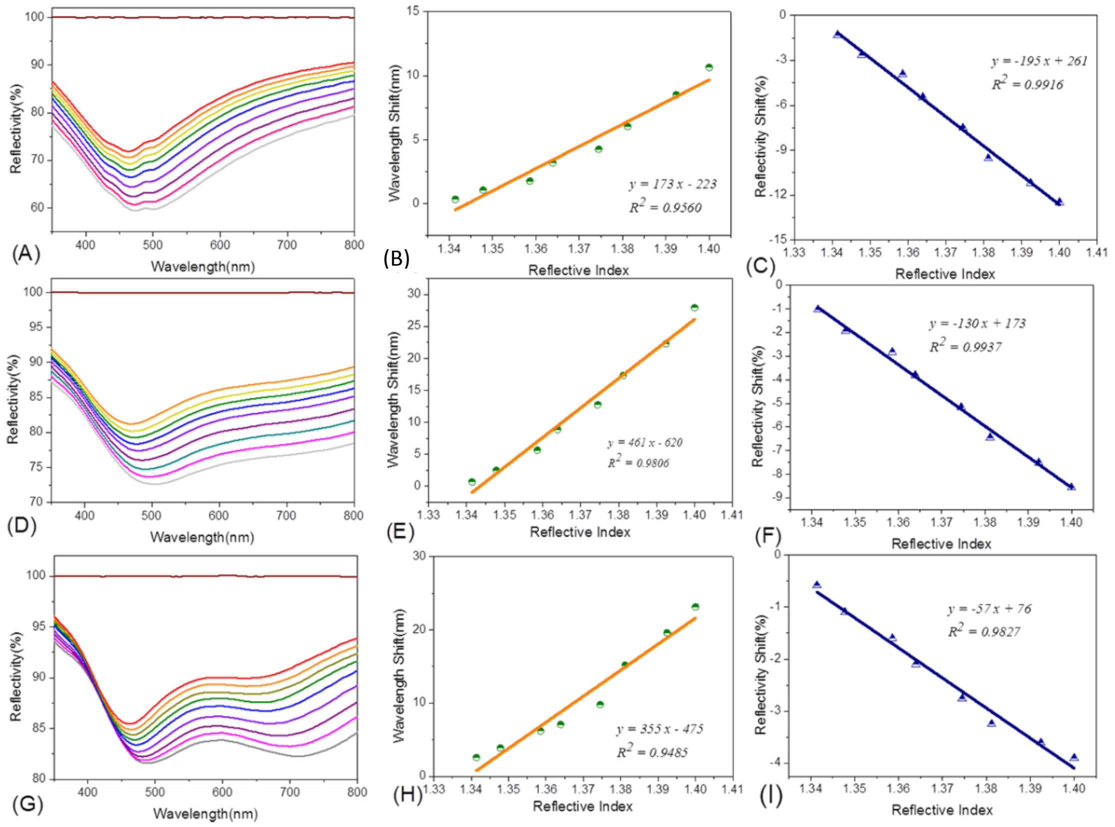

Figure S2. Refractive index sensitivities of the AgNP-based sensors with different coating time of 0.5 h, 1 h, 3 h. (**A**,**D**,**G**) illustrate the reflective spectrum of the AgNP-based sensors with different refractive index solutions; (**B**,**E**,**H**) illustrates the wavelength shift comparison of the AgNP-based sensors with three different coating time; (**C**,**F**,**I**) illustrates the optical intensity shift comparison of the AgNP-based sensors with three different coating time.

© 2015 by the authors; licensee MDPI, Basel, Switzerland. This article is an open access article distributed under the terms and conditions of the Creative Commons Attribution license (http://creativecommons.org/licenses/by/4.0/).
